# Vision and hearing problems and psychosocial outcomes: longitudinal evidence from the German Ageing Survey

**DOI:** 10.1007/s00127-023-02588-9

**Published:** 2023-11-18

**Authors:** André Hajek, Razak M. Gyasi, Benedikt Kretzler, Hans-Helmut König

**Affiliations:** 1 Department of Health Economics and Health Services Research, University Medical Center Hamburg-Eppendorf, Hamburg Center for Health Economics, Martinistr. 52, 20246 Hamburg, Germany; 2 African Population and Health Research Center, Nairobi, Kenya; 3 National Centre for Naturopathic Medicine, Faculty of Health, Southern Cross University, Lismore, NSW Australia

**Keywords:** Hearing impairment, Hearing problems, Visual impairment, Vision problems, Loneliness, Social isolation, Social exclusion, Social contact, Social embeddedness, Depression

## Abstract

**Purpose:**

To examine whether changes in vision and hearing problems are associated with changes in psychosocial outcomes (in terms of depressive symptoms, loneliness, and perceived social isolation).

**Methods:**

We used longitudinal data from the nationally representative German Ageing Survey, which covers individuals aged 43 years and over (wave 6 and wave 7, with 7108 observations and mean age of 67.5 years, SD 10.2 years). The 6-item De Jong Gierveld tool was used to quantify loneliness, the Bude and Lantermann tool was used to quantify perceived social isolation, and the Center for Epidemiologic Studies Depression Scale (15-item version) was used to quantify depressive symptoms. Self-rated problems reading the newspaper due to vision problems and self-rated difficulties recognizing known people on the street due to vision problems were used to quantify vision problems. In addition, self-rated hearing problems on the telephone and self-rated hearing problems in groups of more than four people were used to quantify hearing problems.

**Results:**

Adjusting for various confounders, longitudinal regressions showed that the onset of major vision problems referring to difficulties recognizing people one knows on the street was associated with increases in loneliness (*β* = 0.17, *p* < .01) and depressive symptoms (*β* = 1.90, *p* < 0.05). Moreover, the onset of some vision problems referring to difficulties reading the newspaper was associated with increases in perceived social isolation (*β* = 0.06, *p* < 0.01). Additionally, the onset of some hearing problems in groups of more than four people was associated with increases in depressive symptoms (*β* = 0.43, *p* < 0.05).

**Conclusion:**

Our longitudinal study showed that vision and hearing problems can contribute differently to psychosocial factors. Delaying sensory impairment may result in favorable psychosocial factors in later life.

## Introduction

Nearly 300 million people worldwide are visually impaired. Of those, approximately two out of three are over the age of 50 [1]. This visual impairment is associated with a significant economic and social burden [2, 3]. Additionally, hearing impairment is frequent among older adults [4, 5] and is associated with adverse health consequences [6]. Due to ongoing demographic changes, the number of people suffering from visual impairment and hearing impairment is expected to increase significantly in the upcoming decades [7–9].

Various studies have shown the consequences of sensory impairment on physical and mental health [10–12]. In contrast, the association between sensory impairment and psychosocial factors is not well understood. For example, a previous cross-sectional study showed an association between visual impairment and higher depressive symptoms, as well as higher perceived social isolation [13]. Another prior cross-sectional study demonstrated an association between visual impairment and higher loneliness scores [14]. Additionally, a former cross-sectional study demonstrated an association between hearing impairment and lower mental health [15]. Other recent cross-sectional studies also demonstrated associations between hearing impairment and loneliness or fewer social interactions, respectively [16, 17]. However, thus far, there is very restricted knowledge regarding the associations based on representative longitudinal data. Longitudinal data play a crucial role in addressing the issue of unobserved heterogeneity, which is a significant hurdle in the analysis of observational data, for instance. The ability to better make causal inferences in longitudinal studies arises from having knowledge about the timing of changes.

For instance, based on data from the English Longitudinal study of Ageing, one recent longitudinal study showed a positive longitudinal association between hearing impairment and loneliness as well as social isolation [18]. Another study did not identify a significant longitudinal association between both visual and hearing impairment and loneliness [19]. Due to the limited and inconclusive knowledge based on longitudinal, nationally representative data, the aim of this study was to examine whether changes in vision and hearing problems are associated with psychosocial outcomes (in terms of depressive symptoms, loneliness, and perceived social isolation) using longitudinal data from the German Ageing Survey.

Such knowledge is important because it may stress the importance of delaying sensory impairment—which can contribute to psychosocial factors. This in turn is important because globally about one out of two impairments in hearing are preventable [20]. Moreover, three out of four vision losses are preventable and it also has been shown that several interventions related to eye care are cost effective [21].

## Methods

### Sample

The data came from waves 6 and 7 of a sample of residents aged 40 and over (second half of life; “German Ageing Survey”, abbreviated: DEAS). The Federal Ministry for Family Affairs, Senior Citizens, Women and Youth (BMFSFJ) funded this study, which began in 1996. It was conducted cohort-sequentially, meaning that new baseline samples were added mostly every 6 years [in wave 2 (year 2002); wave 3 (year 2008); wave 5 (year 2014)], while exclusive panel waves were performed in wave 4 (year 2011), wave 6 (year 2017), and wave 7 (November 2020 to February 2021).

A national probability sample was used to select individuals. The DEAS survey covered a variety of topics, including economic factors, health-related factors, family life, or transition to old age. Individuals were interviewed in a first step (in wave 7, only by telephone in the CAPI field which was in accordance with COVID-19 restrictions) on sociodemographic issues and other topics. In a further step, participants were given a questionnaire to complete (also on quite sensitive factors such as psychosocial factors). In wave 7, for example, the response rate was 65%. In this wave, the average interview lasted about 75 min. The most common reasons for the absence of follow-up data were refusal and health limitations. Further details are given by Klaus et al. [22].

We used wave 6 and wave 7 for reasons of data availability in this study. Due to the use of linear fixed effects (FE) regressions in this study, we only included individuals in our analytical sample when they took part in both waves and had within-variations in the variables (i.e., outcome measures, independent variables of interest or time-varying covariates) longitudinally. Consequently, the analytical sample consisted of 7108 observations when depressive symptoms served as outcome measure (this corresponds to 3554 individuals as we used two waves in this study). It should be noted that the analytical sample slightly varied depending on the outcome used. For example, the analytical sample consisted of 7042 observations when perceived social isolation served as outcome measure. These discrepancies can be mainly explained by missing data in the outcome measures.

The Declaration of Helsinki was followed in the conduct of the DEAS study. No ethics statement was required for the DEAS study, as the prerequisites for such a statement were not given (examination of patients, risk for the individuals or lack of information about the aims of the study). The German Center for Gerontology, which is responsible for the DEAS study, did not request an ethics vote based on the recommendation of a DEAS standing advisory committee, which determined that no ethics vote was required.

### Dependent variables

Loneliness, perceived social isolation and depressive symptoms served as outcomes. To quantify loneliness, we used the De Jong Gierveld loneliness tool [23]. This tool has six items (with four levels each). Based on all six items, an average score was computed which ranges from 1 to 4, whereby higher values correspond to higher loneliness levels. In wave 6, Cronbach’s alpha was 0.83 (wave 7: 0.80) and McDonald’s omega was 0.84 (wave 7: 0.81).

Perceived social isolation was measured using Bude and Lantermann’s [24] instrument. There are four items in this instrument (four levels each). Based on these four items, an average score was calculated which also ranges from 1 to 4 and higher values reflect higher perceived social isolation levels. In wave 6, Cronbach’s alpha was 0.87 (wave 7: 0.87) and McDonald’s omega was 0.88 (wave 7: 0.88).

Depressive symptoms were quantified using the Center for Epidemiologic Studies Depression Scale (15-item version) [25]. The sum of all 15 items represents the total score. It ranges from 0 to 45, with higher values reflecting more depressive symptoms. In wave 6, Cronbach’s alpha was 0.86 (wave 7: 0.84) and McDonald’s omega was 0.87 (wave 7: 0.86).

### Independent variables of interest

Individuals self-reported their sensory impairment as follows:

“1. Do vision problems cause you trouble reading the newspaper (possibly even when using a vision aid)?

2. Do you have difficulties recognizing people you know on the street due to vision problems (possibly even when wearing glasses or contacts)?

3. Do you have hearing problems on the telephone (possibly even when using a hearing aid)?

4. Do you have hearing problems in groups of more than four people (possibly even when using a hearing aid)?”.

In all four cases, they could rate their difficulties as follows: 1 = no difficulties, 2 = some difficulties, 3 = major difficulties, 4 = impossible. Due to the low number of cases, we collapsed the last two categories (major difficulties/impossible) into one. Thus, we distinguished between three categories: no difficulties, some difficulties or major difficulties/impossible. Moreover, due to the high correlations between the two variables related to vision impairments (and between the two variables related to hearing impairment), we included only one variable related to visual impairment and one variable related to hearing impairment (first model: first vision impairment variable with the first hearing impairment variable simultaneously in the regression model; second model: second vision impairment variable with the second hearing impairment variable simultaneously in the regression model). Please, see the regression tables for further details.

### Covariates

Grounded on past research [13, 15, 17–19, 26–28], several time-varying sociodemographic, lifestyle-related and health-related covariates were included. Concerning sociodemographic time-varying factors, we included age (years), marital status (five categories: divorced; widowed; single; married, living separated from spouse; married, living together with spouse), and labour force participation (employed; retired; other: not employed).

With regard to lifestyle-related time-varying factors, we included smoking (no, never; no, not anymore; yes, sometimes; yes, daily), alcohol consumption (daily; several times a week; once a week; 1–3 times a month; less often; no, never), and frequency of sports activities (again: daily; several times a week; once a week; 1–3 times a month; less often; no, never) in regression analysis.

Concerning health-related time-varying factors, it was adjusted for self-rated health (1 = very good to 5 = very bad, single item), and a count score for chronic physical conditions. This count score ranges from 0 to 9 and includes the following factors: cardiac and circulatory disorders; bad circulation; joint, bone, spinal and back problems; respiratory problems, asthma, shortness of breath; stomach and intestinal problems; cancer; diabetes; gall bladder, liver or kidney problems; bladder problems).

For descriptive purposes, the time-invariant factors sex (women; men) and education (following the ISCED-97 classification [29], distinguishing between low (0–2), medium (3–4) and high (5–6) education) were used.

### Statistical analysis

First, sample characteristics for the total analytical sample are presented. Thereafter, we conducted multiple linear fixed effects (FE) regressions to examine the link between intraindividual changes in our explanatory variables and intraindividual changes in our outcome measures. It was adjusted for the time-varying sociodemographic, lifestyle-related and health-related covariates which were presented in detail in the previous section.

One key advantage of FE regressions is that they can produce consistent estimates, while relying on weak assumptions [30]. For example, even when time-constant (observed and unobserved) factors are correlated with explanatory factors, FE regressions produce consistent estimates [30]. In contrast, random-effects regressions would yield inconsistent estimates in such a case [30].

Only changes within individuals (i.e., intraindividual changes) over time are exploited in FE regressions such as a change in visual impairment within an individual from wave 6 to wave 7. Intraindividual changes in depressive symptoms, for example, can be examined from wave 6 to wave 7. Time-invariant factors such as sex cannot be included as main effects in linear FE regressions, because they usually do not vary within individuals over time.

The FE technique is not limited by focusing on participants who actually experienced changes in the independent and dependent variables over time; rather, it represents the fact that only a subset of the population actually experienced such changes over time. This corresponds to an average treatment effect on the treated [31].

To determine McDonald’s omega, we used a newly developed tool [32]. The statistical significance was determined as *p* value of < 0.05 in this study. Stata 16 was used for statistical analyses (Stata Corp., College Station, Texas).

## Results

### Sample characteristics

For the pooled analytical sample (*n* = 7108 observations, when depressive symptoms are used as outcome), sample characteristics are given in Table 1. Average age was 67.5 years (SD 10.2 years; 43–98 years) and 50.9% of the individuals were female. Average loneliness score was 1.8 (SD 0.5), average perceived social isolation score was 1.6 (SD 0.6), and average depressive symptoms score was 6.2 (SD 5.7). Moreover, for example, “some difficulties” in the variables related to sensory impairment were present as follows: 6.7% (vision problems referring to difficulties recognizing people you know on the street), 15.5% (vision problems referring to difficulties reading the newspaper), 13.7% (hearing problems on the telephone), 24.3% (hearing problems in groups of more than four people). Further details are shown in Table 1.

**Table 1:** Sample characteristics for the analytical sample (wave 6 and wave 7, pooled, *n* = 7,108 observations)

Variables	Mean (SD)/*N* (%)
Age	67.5 (10.2)
Sex	
Men	3492 (49.1)
Women	3616 (50.9)
Marital status	
Married, living together with spouse	5034 (70.8)
Married, living separated from spouse	77 (1.1)
Divorced	678 (9.5)
Widowed	885 (12.5)
Single	434 (6.1)
Educational level	
Low education	268 (3.8)
Medium education	3296 (46.4)
High education	3544 (49.9)
Employment status	
Employed	2244 (31.6)
Retired	4462 (62.8)
Other: not employed	402 (5.7)
Alcohol intake	
Daily	928 (13.1)
Several times a week	1913 (26.9)
Once a week	1131 (15.9)
1–3 × a month	849 (11.9)
Less often	1596 (22.5)
Never	691 (9.7)
Smoking behavior	
Yes, daily	728 (10.2)
Yes, occassionally	222 (3.1)
No, not anymore	2679 (37.7)
No, never	3479 (48.9)
Frequency of sports activities	
Daily	708 (10.0)
Several times a week	2134 (30.0)
Once a week	1269 (17.9)
1–3 × a month	413 (5.8)
Less often	777 (10.9)
Never	1807 (25.4)
Self-rated health	2.4 (0.8)
Number of chronic conditions	1.9 (1.5)
Vision problems referring to difficulties recognizing people you know on the street	
No difficulties	6546 (92.1)
Some difficulties	478 (6.7)
Major difficulties/impossible	84 (1.2)
Vision problems referring to difficulties reading the newspaper	
No difficulties	5853 (82.3)
Some difficulties	1103 (15.5)
Major difficulties/impossible	152 (2.1)
Hearing problems on the telephone	
No difficulties	6012 (84.6)
Some difficulties	975 (13.7)
Major difficulties/impossible	121 (1.7)
Hearing problems in groups of more than four people	
No difficulties	4964 (69.9)
Some difficulties	1725 (24.3)
Major difficulties/impossible	411 (5.8)
Loneliness	1.8 (0.5)
Perceived social isolation	1.6 (0.6)
Depressive symptoms	6.2 (5.7)

### Regression analysis

The results of linear FE regressions with loneliness, perceived social isolation and depressive symptoms as outcomes are shown in Table 2. In all FE regressions, it was adjusted for the time-varying covariates age, family situation, employment status, smoking behavior, alcohol consumption, sports activities, self-rated health and the number of physical illnesses. We also checked the number of transitions in the independent variables. For example, in the variable referring to hearing problems in groups of more than 4 people, 281 intraindividual transitions (from wave 6 to wave 7) were present from “no difficulties” to “some difficulties” and 72 transitions were present from “some difficulties” to “major difficulties/impossible”. In contrast, in the variable referring to hearing problems in groups of more than 4 people, for example, 2,168 individuals remained in the “no difficulties” group. However, worth repeating, individuals not reporting changes over time do not provide to the beta-coefficients of the respective variables.

**Table 2:** Determinants of psychosocial outcomes

Independent variables	(1)	(2)	(3)	(4)	(5)	(6)
	Loneliness	Loneliness	Perceived social isolation	Perceived social isolation	Depressive symptoms	Depressive symptoms
Vision problems referring to difficulties reading the newspaper:- Some difficulties (Reference: No difficulties)	0.02		0.06**		0.34	
	(0.02)		(0.02)		(0.21)	
- Major difficulties/impossible	0.02		0.05		0.69	
	(0.04)		(0.05)		(0.61)	
Hearing problems on the telephone:- Some difficulties (Ref.: No difficulties)	− 0.01		0.01		− 0.19	
	(0.02)		(0.02)		(0.24)	
- Major difficulties/impossible	0.02		0.05		− 0.24	
	(0.04)		(0.06)		(0.65)	
Vision problems referring to difficulties recognizing people you know on the street:- Some difficulties (Reference: No difficulties)		0.01		0.00		0.32
		(0.02)		(0.03)		(0.34)
- Major difficulties/impossible		0.17**		0.12		1.90*
		(0.05)		(0.08)		(0.91)
Hearing problems in groups of more than four people:- Some difficulties (Reference: No difficulties)		0.00		0.01		0.43*
		(0.02)		(0.02)		(0.21)
- Major difficulties/impossible		0.04		0.06 +		0.54
		(0.03)		(0.04)		(0.44)
Time-varying covariates	✓	✓	✓	✓	✓	✓
Constant	0.68***	0.68***	0.60**	0.63**	− 0.30	− 0.45
	(0.17)	(0.17)	(0.22)	(0.22)	(2.32)	(2.32)
Observations	7080	7070	7042	7034	7108	7098
Number of individuals	3540	3535	3521	3517	3554	3549
*R*^2^	0.02	0.03	0.02	0.02	0.08	0.08

Results of multiple linear FE regressions

Unstandardized beta-coefficients are displayed, robust standard errors in parentheses; ***p < 0.001, **p < 0.01, *p < 0.05, + *p* < 0.10

Time-varying covariates include age, family situation, employment status, smoking behaviour, alcohol consumption, sports activities, self-rated health and the number of physical illnesses

Increases in *loneliness* were significantly associated with changes from “no difficulties” to “major difficulties/impossible” in vision problems referring to difficulties recognizing people one know on the street (*β* = 0.17, *p* < 0.01), whereas they were not associated with changes in the other variables related to sensory impairment. Increases in *perceived social isolation* were significantly associated with changes from “no difficulties” to “some difficulties” in vision problems referring to difficulties reading the newspaper (*β* = 0.06, *p* < 0.01), whereas they were not associated with changes in the other variables related to sensory impairment. Lastly, increases in *depressive symptoms* were significantly associated with changes from “no difficulties” to “major difficulties/impossible” in vision problems referring to difficulties recognizing people one know on the street (*β* = 1.90, *p* < 0.05) and changes from “no difficulties” to “some difficulties” in hearing problems in groups of more than four people (*β* = 0.43, *p* < 0.05), whereas they were not associated with changes in the other variables related to sensory impairment.

In a sensitivity analysis, the main model was extended by adding year (period) effects. However, our key findings remained virtually the same in terms of effect sizes and significance.

## Discussion

Using data from a longitudinal nationally representative sample, our purpose was to investigate the association between sensory impairment and psychosocial factors over time. Longitudinal regressions revealed that the onset of major vision problems referring to difficulties recognizing people one know on the street was associated with increases in loneliness and depressive symptoms. Moreover, the onset of some vision problems referring to difficulties reading the newspaper was associated with increases in perceived social isolation. Additionally, the onset of some hearing problems in groups of more than four people was associated with increases in depressive symptoms. Our current longitudinal and representative study markedly extends our current knowledge mainly based on cross-sectional studies in this research area [13–17].

It may be worth noting that most of the previous studies (e.g., [3, 33]) focused on overall self-rated sensory impairment rather than specific problems (e.g., problems referring to difficulties reading the newspaper or hearing problems in groups of more than four people). Due to these discrepancies in the assessment of sensory impairments (and the longitudinal design), it is rather challenging to compare our current results with prior research.

The consequences of major vision problems referring to difficulties recognizing people one know on the street for psychosocial factors (i.e., loneliness and depressive symptoms) may be mainly attributed to reduced social contacts [33]. More precisely, not being able to recognize people could, among other things, lead to a reduction in appropriate conversations (e.g., a chat when meeting a friend while shopping) [34]. This could increase feelings of loneliness. Moreover, the association can also be attributed to the fact that a decreased ability to recognize familiar people can lead to anxiety, which in turn leads to increased levels of loneliness.

It should be noted that newspapers in Germany usually convey current events locally, nationally or even worldwide. Difficulties in reading the newspaper due to vision problems may contribute to no longer reading newspapers at all. This could reinforce the feeling of being out of touch with current events locally/in Germany or in the world. This in turn could reinforce the feeling of being excluded from the society [35]. Inadequate newspaper reading can leave individuals without useful daily information that they may want to share with colleagues and peers, thereby affecting interpersonal relationships—which can contribute to social isolation in later life.

Hearing problems in groups of more than four people can lead to not being able to follow conversations (in larger groups) properly. This can lead to avoidance of larger groups. For example, such individuals may avoid activities in clubs or volunteer activities. Such avoidance may reflect a decline in social activities [36]. Ultimately, this may contribute to increases in depressive symptoms [37].

When interpreting our current findings, it is important to keep some strengths and limitations in mind. To begin, a large, nationally representative sample was employed. Moreover, longitudinal data were used and the problem of unobserved heterogeneity—which reflects a key challenge when dealing with observational data—was addressed by using FE regressions. Furthermore, the outcomes (i.e., depressive symptoms, loneliness and perceived social isolation) were assessed using well-established and reliable tools.

Prior research showed differences between self-reported measures for sensory impairments and clinical assessments (e.g., [28]). Nevertheless, we assume that self-rated problems in sensory impairment could more accurately reflect the character of sensory impairments for one’s own life [27]. It should be noted that a small selection bias has been identified in the DEAS study [22].

## Conclusion

Our longitudinal study showed that vision and hearing problems can contribute differently to psychosocial factors. Delaying sensory impairment may result in favorable psychosocial factors in later life.

## Data Availability

The data used in this study are third-party data. The anonymized data sets of the DEAS (1996, 2002, 2008, 2011, 2014, 2017, 2020, 2020/2021) are available for secondary analysis. The data have been made available to scientists at universities and research institutes exclusively for scientific purposes. The use of data is subject to written data protection agreements. Microdata of the German Ageing Survey (DEAS) are available free of charge to scientific researchers for non-profitable purposes. The FDZ-DZA provides access and support to scholars interested in using DEAS for their research. However, for reasons of data protection, signing a data distribution contract is required before data can be obtained. For further information on the data distribution contract, please see https://www.dza.de/en/research/fdz/access-to-data/formular-deas-en-english (Accessed on 21 March 2023).
